# Complete Transection of the Abdominal Wall Secondary to Being a Rear Seat-Belted Passenger

**DOI:** 10.1155/2024/4335543

**Published:** 2024-06-27

**Authors:** Jamshed Zuberi, Yousef Masoudpoor, Severija Saladziute, Emma Morel, Daniel Garcia, Diya Khetan, Erem Tutal, Natasha Becker, Peter Park, Stephanie Bonne

**Affiliations:** Hackensack University Medical Center, Hackensack, NJ 07601, USA

## Abstract

Garrett and Braunstein introduced the concept of the “seat belt sign” in motor vehicle collision (MVC) victims. They defined this as abdominal wall bruising from a lap belt. These signs of trauma are not uncommon. However, “seat belt syndrome,” a pattern of musculoskeletal and internal organ injuries resulting from deceleration forces exerted by the safety device is rarely seen. Here, we illustrate a case of traumatic closed rupture of the rectus abdominis muscle secondary to seat belt injury. This potential injury is important to recognize and our case will illustrate the need for careful imaging review and clinical assessment to identify associated intra-abdominal injuries.

## 1. Introduction

Seat belt syndrome, a rare pattern of musculoskeletal and internal organ injuries caused by deceleration forces from safety devices, is often overlooked [1, 2]. We present a patient with traumatic closed rupture of the bilateral rectus abdominis muscles and transversalis muscles with associated intra-abdominal injury secondary to seat belt injury. This case emphasizes the importance of careful imaging and clinical assessment for detecting associated intra-abdominal injuries [3, 4].

## 2. Case Presentation

**Figure 1:**
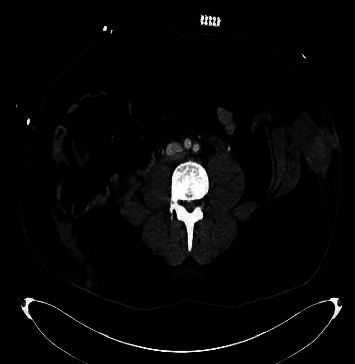
Abdominal CT. Transection of the lateral abdominal wall muscles along with herniation of bowel contents.

**Figure 2:**
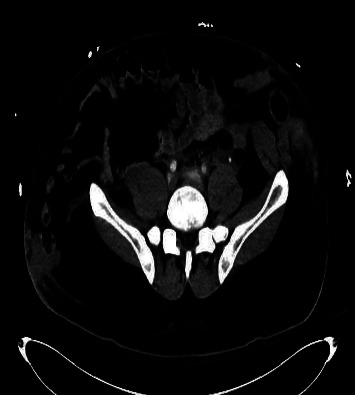
Abdominal CT. Bilateral herniation of abdominal viscera.

**Figure 3:**
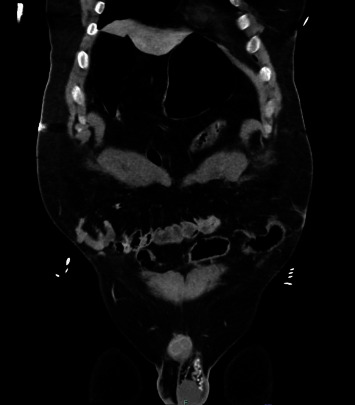
Abdominal CT. Bilateral transection of rectus abdominis musculature (arrows) with resultant bowel herniation.

**Figure 4:**
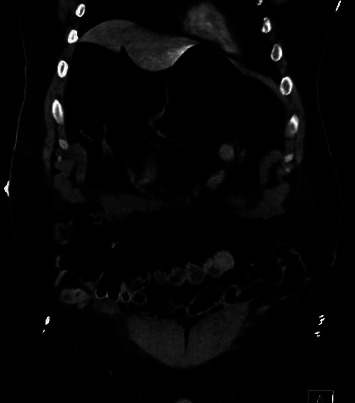
Abdominal CT. Absence of the rectus muscles with resultant bowel herniation.

A 29-year-old male with obesity (BMI 32) and type 1 diabetes presented to the emergency department following a high-speed motor vehicle collision (MVC) as a restrained rear passenger. He complained of neck, chest, and lower abdominal pain on initial assessment, with tachycardia (HR 119) and hypotension (BP 102/56 mmHg). His abdominal exam did not demonstrate peritoneal signs, but seatbelt-patterned abrasions were evident on the neck, chest, and lower abdomen. The patient responded positively to resuscitation, and subsequent X-rays of the chest and pelvis revealed no injuries. CT scans revealed complete absence of the rectus muscles below the umbilicus with bowel herniation along with fractures of ribs and spinal transverse processes (Figures 1, 2, 3, and 4).

**Figure 5:**
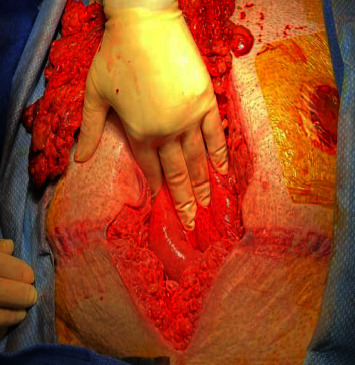
Intraoperative photograph showing the seat belt sign.

The patient underwent exploratory laparotomy, revealing extensive abdominal muscle transection and colon devascularization with perforation (Figure 5). Damage control surgery was performed to ensure viability of the bowel because of the extensive devascularization caused by the crush injury, followed by a second look laparotomy during which Hartman's type colostomy was matured, and primary abdominal wall repair was performed. Post-op course was complicated by prolonged respiratory failure. Ultimately the patient was discharged to a rehabilitation facility 1 month following the initial presentation.

## 3. Discussion

This case highlights the challenges of treating closed traumatic rupture of abdominal wall musculature [5], which is often complicated by patient factors and associated injuries [6]. While CT imaging is essential for diagnosing blunt abdominal trauma [7], clinical presentation should guide medical and surgical decision making [8, 9]. Early detection of mesenteric injuries is crucial, as they may not immediately appear on imaging. Furthermore, in the setting of acute bowel injury and perforation, the use of implantable mesh is not ideal which complicates reconstruction of the abdominal wall [10].

## 4. Conclusion

Cases of intra-abdominal musculature transection due to deceleration seat-belt injuries often present with fairly unremarkable initial exams and imaging [11]. Although rare, traumatic abdominal wall hernias are often associated with significant intra-abdominal injuries, and repair can present significant technical challenges [12]. This report emphasizes the importance of maintaining a low threshold for operative exploration in such cases to enhance patient outcomes [13].
